# Fecal continence outcomes are associated with the type, height, and stage procedure of ileal pouch-anal anastomosis

**DOI:** 10.1007/s00384-020-03626-7

**Published:** 2020-05-30

**Authors:** Jara E. Jonker, Hendrik S. Hofker, Monika Trzpis, Paul M. A. Broens

**Affiliations:** 1 Department of Surgery, Anorectal Physiology Laboratory, University of Groningen, University Medical Center Groningen, Groningen, The Netherlands; 2 Department of Surgery, Division of Abdominal Surgery , University of Groningen, University Medical Center Groningen, Groningen, The Netherlands; 3 Department of Surgery, Division of Pediatric Surgery, University of Groningen, University Medical Center Groningen, Groningen, The Netherlands

**Keywords:** Ileal pouch-anal anastomosis, Fecal continence, Stapled anastomosis, Hand-sewn anastomosis, Quality of life, Stage procedures

## Abstract

**Purpose:**

This study aims to analyze the quality of life in patients with an ileal pouch-anal anastomosis (IPAA) and to investigate the association between height and type of the anastomosis, the number of stage procedures and age, and the fecal continence outcomes.

**Methods:**

This is a cross-sectional retrospective study in patients who had undergone IPAA between 1992 and 2016 (*N* = 133). We sent questionnaires to 102 eligible patients (64% response rate). We used the Wexner score to assess fecal incontinence: 0 = no incontinence to 20 = complete incontinence. We used RAND-36 to measure quality of life.

**Results:**

Patients who underwent mucosectomy with hand-sewn anastomoses (*n* = 11, 17%) had significantly higher median Wexner scores than patients with stapled anastomoses (10 versus 3, *P =* 0.003). Lower anastomoses correlated significantly with increasing Wexner scores (*r* = − 0.468, *P <* 0.001). Quality of life of incontinent patients was diminished. Patients who were older at the time of IPAA surgery had higher Wexner scores (*P* = 0.004), while the time between surgery and questionnaire did not influence their Wexner scores (*P* = 0.810). Considering the stage procedures, multiple linear regression showed that the two-stage procedure without diverting ileostomy was significantly associated with higher Wexner scores (*B* = 0.815, *P =* 0.02), adjusted for sex (*P =* 0.008) and anastomosis type (*P =* 0.002). The three-stage procedure showed equally low complications and anastomotic leakage rates.

**Conclusion:**

Mucosectomy with more distal, hand-sewn anastomosis and increasing age at IPAA surgery was associated with poorer fecal continence outcomes. The three-stage procedure appears to give the best fecal continence results without increasing complications. Furthermore, incontinence reduced patient’s quality of life.

## Introduction

Fecal incontinence is a devastating condition that significantly impairs the quality of life [1]. In patients who undergo proctocolectomy with an ileal pouch-anal anastomosis (IPAA), fecal continence outcomes are under debate [2, 3]. It is the procedure of choice for many patients who require surgery for ulcerative colitis or familial adenomatous polyposis [3, 4]. It is known that these patients have a better quality of life after restorative proctocolectomy [5]. Nevertheless, there are only a few studies that report on the quality of life of IPAA patients in relation to functional outcomes [6–8].

Two types of anastomoses during proctocolectomy with IPAA have been described, namely mucosectomy of the distal rectal tissue followed by a hand-sewn anastomosis at the dentate line and a stapled anastomosis with some rectal cuff retained [9]. During the last decades, the number of stapled IPAA has increased steadily [9, 10]. Stapled anastomoses are quicker and supposedly result in better outcomes in terms of fecal continence, possibly because of the preservation of the anal canal/anal transition zone [9–12]. Another study, however, reported similar clinical outcomes in patients after hand-sewn and stapled anastomoses, and still no consensus exists about the ideal height of the stapled anastomosis [13].

Proctocolectomy with IPAA can be performed in different stage procedures, namely in a one-, two-, or three-stage procedure [14]. Fewer procedures could mean lower costs, a shorter stay in hospital, and less unpleasant temporary stomas [15, 16]. Nevertheless, clinical outcomes for the procedure are also important. To date, no consensus exists in the literature as to which of the stage procedures provides the best clinical outcomes after IPAA surgery [14–22].

The aim of this study was to analyze fecal continence outcomes in patients who had undergone proctocolectomy with IPAA with regard to their quality of life. Furthermore, we aim to determine whether mucosectomy with hand-sewn anastomosis versus stapled anastomosis, as well as the height of the IPAA anastomosis influences patients’ fecal continence outcome. In addition, we aim to compare fecal continence outcomes of patients who had undergone one-, two-, or three-stage procedures during IPAA surgery and compare postoperative complications.

## Methods

### Patient selection

**Fig. 1:**
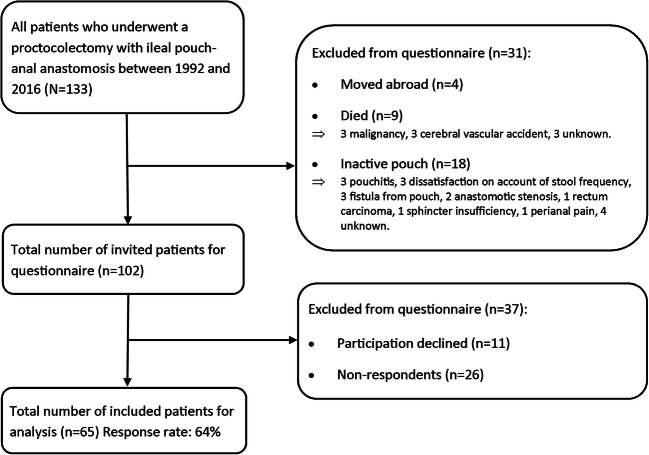
Flowchart of the total number of patients included and the number of patients included in the questionnaire analysis

This study was a cross-sectional questionnaire study. Retrospectively, we reviewed the clinical records of all patients who had undergone a proctocolectomy with IPAA at the University Medical Center Groningen, the Netherlands, between January 1992 and December 2016 (*N* = 133), with a median follow-up of 10 years. Subsequently, we sent the validated Groningen DeFeC questionnaire [23] to these patients. Figure 1 describes the patient selection process with regard to the questionnaire and explanation of exclusion. Finally, 65 of the 102 patients completed the questionnaire (64%), and their data were therefore available for further analysis.

This study was conducted in compliance with our local medical ethics review board. All patients who participated gave their written informed consent.

### Parameters

Retrospectively, we collected data on the characteristics of the proctocolectomy with IPAA. At our hospital, mucosectomy was always followed by a hand-sewn anastomosis and, therefore, will hereafter be referred to as “hand-sewn anastomosis.” Stapled anastomosis without a mucosectomy will be referred to as “stapled anastomosis.” An inactive pouch was defined as removal of the pouch or in case an ileostomy had been created and the pouch was no longer in use. The height of the anastomosis above the dentate line was registered according to the surgery record, usually measured by direct digital palpation. Anastomotic leakage was defined if confirmed by endoscopy and/or radiography imaging or surgery and registered accumulatively for all different stage procedures. Rectal eversion was defined as the eversion of the distal rectum through the anus. This technique was sometimes performed to facilitate the creation of the anastomosis.

Patients were treated by performing either a one-, two-, or three-stage procedure. During a one-stage procedure, a proctocolectomy with IPAA is performed during one surgical procedure. In the case of a two-stage procedure, first proctocolectomy with IPAA with diverting ileostomy is created, followed by a second procedure during which reversal of the ileostomy and gastrointestinal continuity is restored. Another two-stage procedure option is to first perform a subtotal colectomy with end ileostomy, followed by a proctectomy IPAA without diverting ileostomy. During a three-stage procedure, first a subtotal colectomy and temporary end ileostomy is performed, then the IPAA is created with diverting ileostomy, and finally, the ileostomy is reversed and gastrointestinal continuity restored. At our hospital, the policy regarding the number of stages has always been rather conservative. In our clinical experience, where medical therapy-resistant ulcerative colitis is suffered by the majority of our patients, standard practice is to perform the three-stage procedure. The number of stages was determined preoperatively and performed accordingly in 120 patients. Sometimes, it was changed during surgery based on the surgeon’s clinical appraisal of the situation. In five patients, the number of stages was upgraded on account of a complex procedure, and in four patients, it was downgraded because a less complex procedure was actually required than had been expected. In another three patients, the stage procedure was downgraded because insufficient length of intestine was available for creating the diverting ileostomy proximal of the pouch. In one patient, no data were available regarding the determination of the number of stages. Twelve different surgeons performed the IPAAs, and they were equally divided among the stage procedures.

The Clavien-Dindo classification of surgical complications was used for each procedure if the complication occurred within 30 days postoperatively [24]. Because patients who underwent more stage procedures concomitantly had more surgeries, we analyzed the accumulated complications of all operations.

Retrospectively, we collected the date and reason of pouch removal or inactivation or the last date available in the patients’ medical records. Furthermore, we registered whether patients had developed pouchitis, defined as such if confirmed by endoscopic imaging and requiring treatment.

### Questionnaires

We sent the validated DeFeC questionnaire to all eligible patients [23]. Fecal incontinence was defined according to the Rome IV criteria for fecal incontinence [25]. Furthermore, the Jorge-Wexner incontinence score, abbreviated as Wexner score, was extracted from the questionnaire to evaluate the severity of fecal incontinence [26]. The Wexner score ranges from 0 to 20, where 0 indicates no incontinence and a score of 20 indicates that a patient is completely incontinent.

We used the RAND-36 quality-of-life assessment, which was validated for the Dutch population [27, 28].

The patients were asked to complete the online questionnaire or, if they preferred, we sent them a paper version by mail. The online version was designed in such a way that it was mandatory to answer all the questions. Technically, it was only possible for the respondents to return the questionnaire if they had answered all the questions. Of all 65 respondents, 58 (89%) completed the online version and 7 (11%) preferred a paper version, which they returned to us by mail. In three cases, a few questions had remained unanswered. These were completed by the researcher during a telephone interview with the respondent in question.

### Statistical analyses

Data were analyzed with SPSS 23.0 for Windows (IBM SPSS Statistics, IBM Corporation Armonk, NY). Values as numbers (percentages), means ± standard deviations, or as medians (ranges) are reported. Fisher’s exact test was used to compare proportions. Kaplan-Meier analyses were performed to estimate pouch and pouchitis-free survival rates. Spearman’s test was used for correlations. The Independent samples *t* test and the Mann-Whitney *U* test was performed to compare two groups in the case of normal distributed and non-normal distributed data, respectively. Logistic regression was performed, reported as odds ratio (OR), and 95% confidence interval (95% CI). Square root of the Wexner score was used to transform it to a normal distribution. Univariate and multiple linear regression analyses were performed. For the sake of illustration, the quality-of-life scores are presented as means. The level of statistical significance was set at *P* < 0.05.

Figures were generated using either GraphPad Prism 7.02 for Windows, GraphPad Software, La Jolla California, USA or Microsoft Office Publisher 2010.

## Results

### Patients’ characteristics

**Fig. 2:**
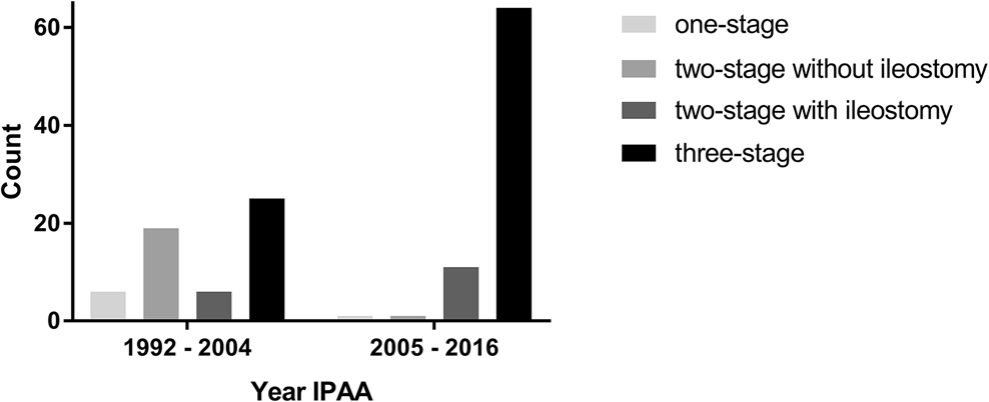
Count of the different stage procedures before 2005 and from 2005 to 2016. Abbreviation: IPAA, ileal pouch-anal anastomosis

Table 1 lists demographic and surgical characteristics including complications of the total cohort of IPAA patients from 1992 to 2016. They were divided into two groups, the responders to the questionnaire and the non-responders, and compared. Median time to follow-up did not differ between the two groups (10 versus 11 years, *P =* 0.57). The number of stages before 2005 and from 2005 to 2016 is shown in Fig. 2. Out of 12 patients with familial adenomatous polyposis, 10 (91%) underwent mucosectomy, and out of 112 patients with active inflammatory bowel disease, 12 (11%) underwent mucosectomy. Of the eight patients with S-pouches, seven (88%) underwent mucosectomy; whereas, of the 117 patients with J-pouches, 18 (15%) underwent mucosectomy. The percentages of anastomotic leakage were 14% for the one-stage procedure, 0% for two-stage procedure with diverting ileostomy, 10% for two-stage procedure without ileostomy, and 7% for the three-stage procedure and were not significantly different (*P =* 0.51). Overall, 1-, 5-, and 10-year pouch survival was estimated at 96.9, 91.0, and 86.0%, respectively. Furthermore, 1-year pouchitis-free survival was estimated at 92.5%, for 5-year pouchitis-free survival, this was 74.8%, and 10-year pouchitis-free survival was estimated at 59.8%.

**Table 1:** Comparison between patients who completed the questionnaire and patients for whom no questionnaire was available

	Total group (*N* = 133)		Questionnaire (*n* = 65)		No questionnaire (*n* = 68)		*P* value
	*n*		*n*		*n*		
Demographics							
Sex	133		65		68		
Men		77 (58%)		40 (62%)		37 (54%)	0.48
Women		56 (42%)		25 (38%)		31 (46%)	
Mean age at IPAA (years)	133	36 ± 13	65	40 ± 12	68	32 ± 13	**< 0.001**
Mean BMI (min-max)	94	25 ± 4.2	59	25 ± 3.0	35	24 ± 5.7	0.50
Surgical indication	133		65		68		
FAP		12 (9%)		3 (5%)		9 (13%)	0.26
Active IBD		112 (84%)		56 (86%)		56 (82%)	
IBD dysplasia		4 (3%)		3 (5%)		1 (1%)	
Other		5 (4%)		3 (5%)		2 (3%)	
Surgical characteristics							
Strategy	133		65		68		
1-stage procedure		7 (5%)		3 (5%)		4 (6%)	0.86
2-stage procedure		37		20		17	
With ileostomy		17 (13%)		10 (15%)		7 (10%)	
Without ileostomy		20 (15%)		10 (15%)		10 (15%)	
3-stage procedure		89 (67%)		42 (65%)		47 (69%)	
Technique proctocolectomy with IPAA	128		63		65		
Open		90 (70%)		44 (70%)		46 (71%)	1.00
Laparoscopic		38 (30%)		19 (30%)		19 (29%)	
Median distance anastomosis from dentate line in cm (min-max)	101	2.0 (0.0–5.5)	51	2.0 (0.0–5.5)	50	2.0 (0.0–5.0)	0.56
Anastomosis technique	125		63		62		
Hand-sewn		25 (20%)		11 (17%)		14 (23%)	0.51
Stapled		100 (80%)		52 (83%)		48 (77%)	
Type of pouch	131		64		67		
J-pouch		120 (92%)		61 (95%)		59 (88%)	0.21
S-pouch		11 (8%)		3 (5%)		8 (12%)	
Median length pouch in cm (min-max)	79	15 (8–21)	37	15 (8.0–20)	42	15 (10–21)	0.35
Rectum eversion	124		62		62		
Yes		35 (28%)		17 (27%)		18 (29%)	1.00
No		89 (72%)		45 (73%)		44 (71%)	
Complications IPAA							
Clavien Dindo grades	132		65		67		
Zero		56 (42%)		29 (45%)		27 (40%)	0.83
I		35 (27%)		16 (25%)		19 (28%)	
II		19 (14%)		11 (17%)		8 (12%)	
IIIa		2 (2%)		1 (2%)		1 (2%)	
IIIb		20 (15%)		8 (12%)		12 (18%)	
Anastomotic leakage	127	9 (7%)	64	4 (6%)	63	5 (7%)	0.74
During pouch surgery		4 (3%)		1 (2%)		3 (4%)	
During ileostomy surgery		5 (4%)		3 (5%)		2 (3%)	

*IPAA*, ileal pouch-anal anastomosis; *FAP*, familial adenomatous polyposis; *IBD*, inflammatory bowel disease

Of all 116 patients with inflammatory bowel disease, 111 (96%) were verified by a pathologist as suffering from ulcerative colitis. In two patients, this diagnosis was later changed to Crohn’s disease. In four cases, it proved difficult, at later stage, to distinguish between ulcerative colitis and Crohn’s disease because histologically the image of the cells may fit either.

### Fecal continence outcomes

**Fig. 3:**
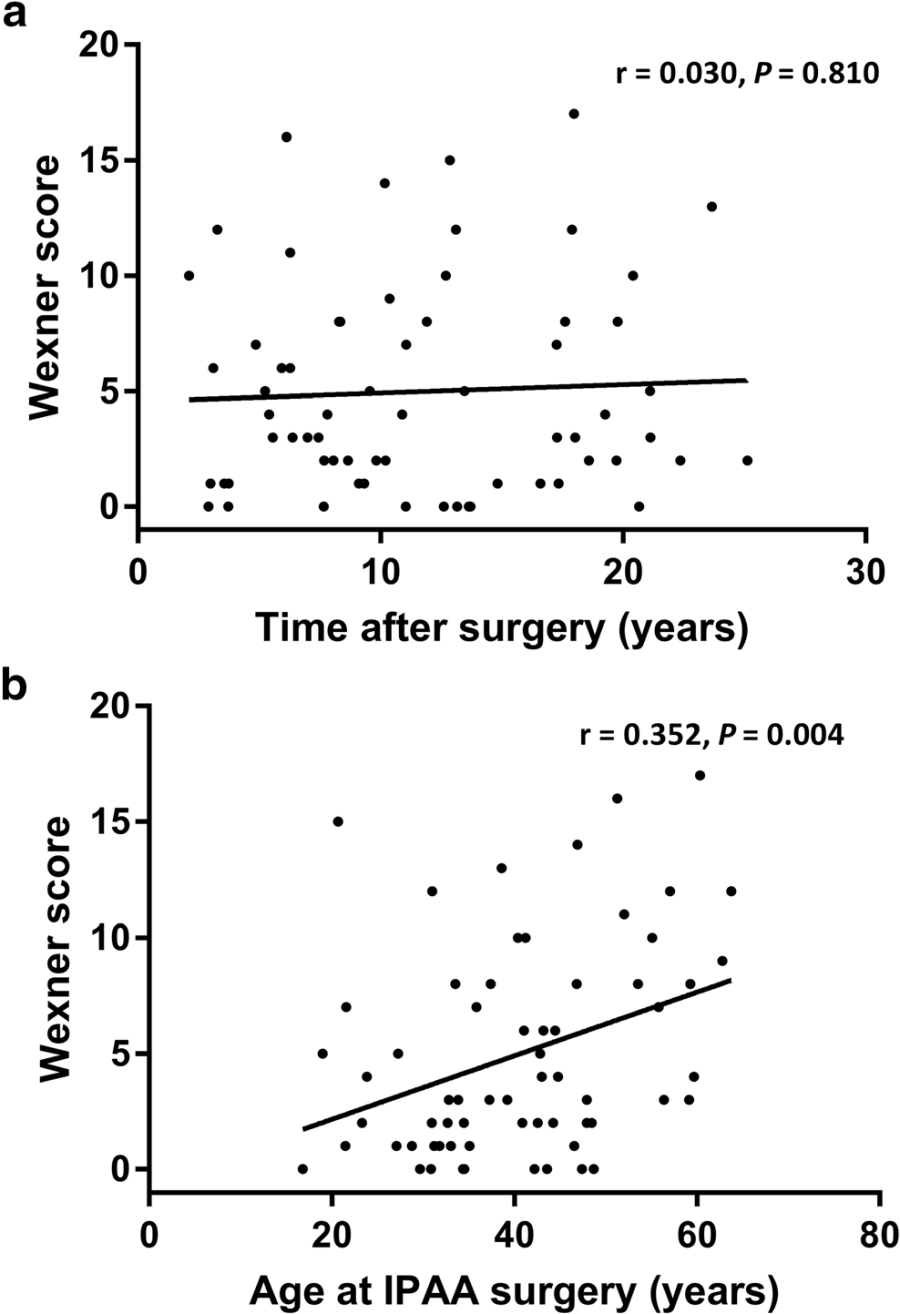
**a** Scatterplot of Wexner scores and the time between surgery and completing the questionnaire in years. **b** Scatterplot of Wexner score and the age at IPAA surgery in years. Abbreviation: IPAA, ileal pouch-anal anastomosis

Out of the 65 patients who had completed the questionnaire, we found that 24 (37%) experienced fecal incontinence. Women tended towards having more fecal incontinence compared with men (13/25 (52%) versus 11/40 (28%), *P =* 0.07). The patients’ median Wexner score was 3 (0–17). The Wexner score was significantly higher in women compared with men (7 versus 2, *P =* 0.006). We found no correlation between the Wexner score and follow-up time after surgery (*r* = 0.030, *P =* 0.81, Fig. 3a). Nevertheless, increased patients’ age at the time of IPAA surgery correlated significantly with an increasing Wexner score (*r* = 0.352, *P =* 0.004, Fig. 3b). We found no differences between the median Wexner scores of patients who had ever or never experienced pouchitis (3 versus 4, *P =* 0.91) nor in patients who had undergone either laparoscopic or open surgery (4 versus 3, *P =* 0.88).

### Quality of life of IPAA patients

**Fig. 4:**
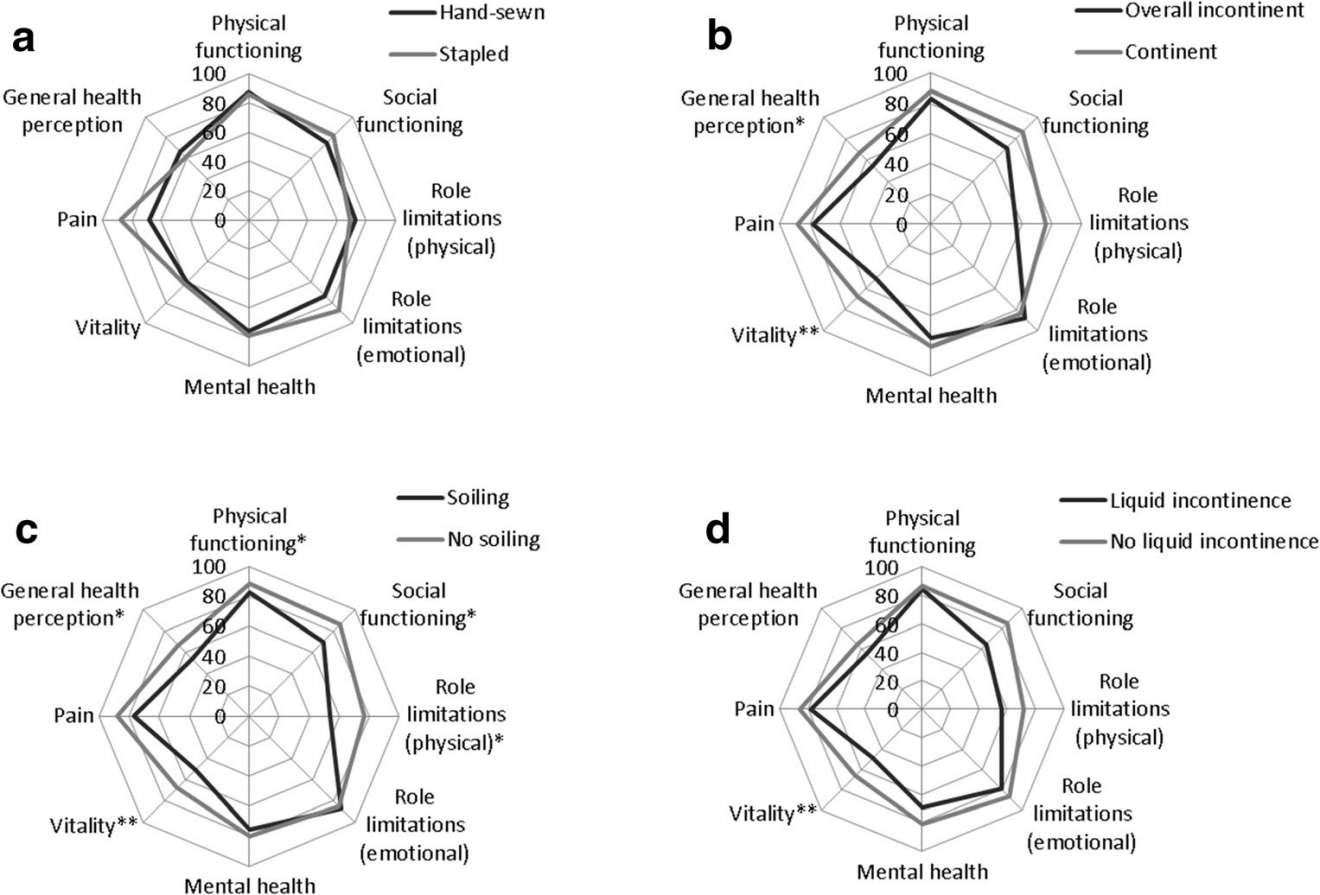
Radar chart of the quality of life of patients with IPAA. Means are given. Statistical significance was shown as follows: *P < 0.05; **P < 0.01. **a** Hand-sewn anastomosis versus stapled anastomosis. **b** Overall incontinent versus continent. **c** Soiling versus no soiling. **d** Liquid stool incontinence versus no liquid stool incontinence

The outcomes on the eight different scales assessing the quality of life of IPAA patients are shown in Fig. 4. There was no statistical difference between the scales for patients with hand-sewn anastomosis compared with stapled anastomosis. Lower outcomes for vitality (*P* = 0.001) and perception of general health (*P* = 0.034) were found in patients who suffered from incontinence in comparison with continent patients. Patients who experienced soiling had significantly lower scales compared with patients who did not experience soiling for physical functioning (*P* = 0.045), social functioning (*P* = 0.041), role limitations (physical) (*P* = 0.041), vitality (*P* = 0.001), and general health perception (*P* = 0.025). Patients who experienced liquid stool incontinence had a lower scale for vitality compared with patients who did not suffer from liquid stool incontinence (*P* = 0.007).

### Association of type and height of the anastomosis with fecal continence outcomes

**Fig. 5:**
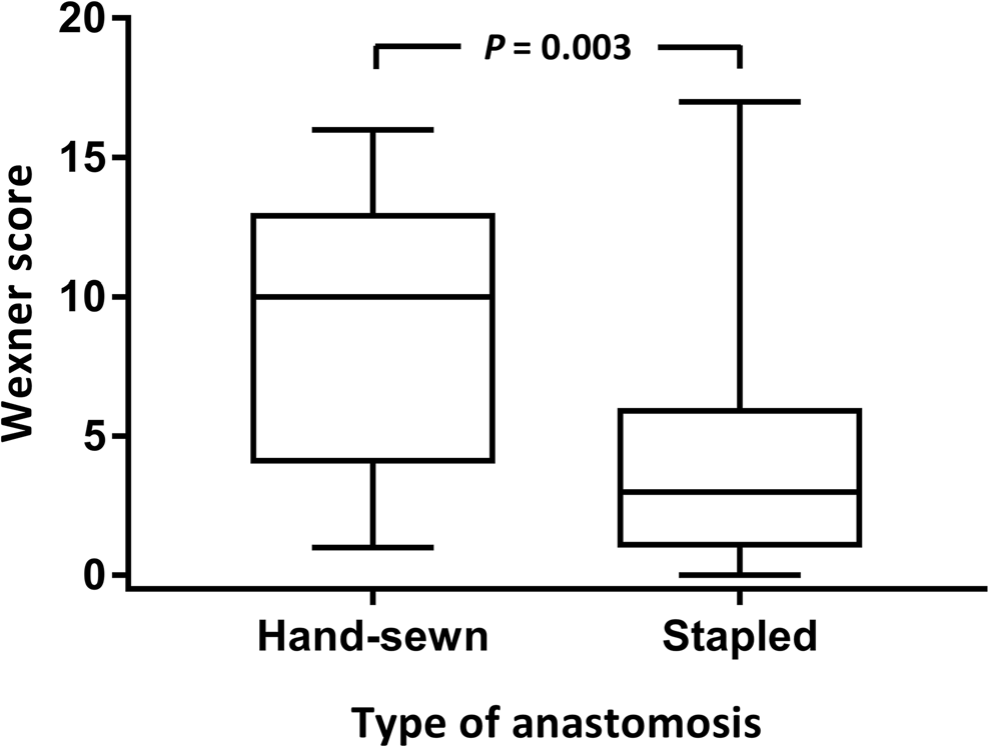
Boxplot of the Wexner score for the group with mucosectomy followed by a hand-sewn anastomosis and the group with a stapled anastomosis without mucosectomy

**Fig. 6:**
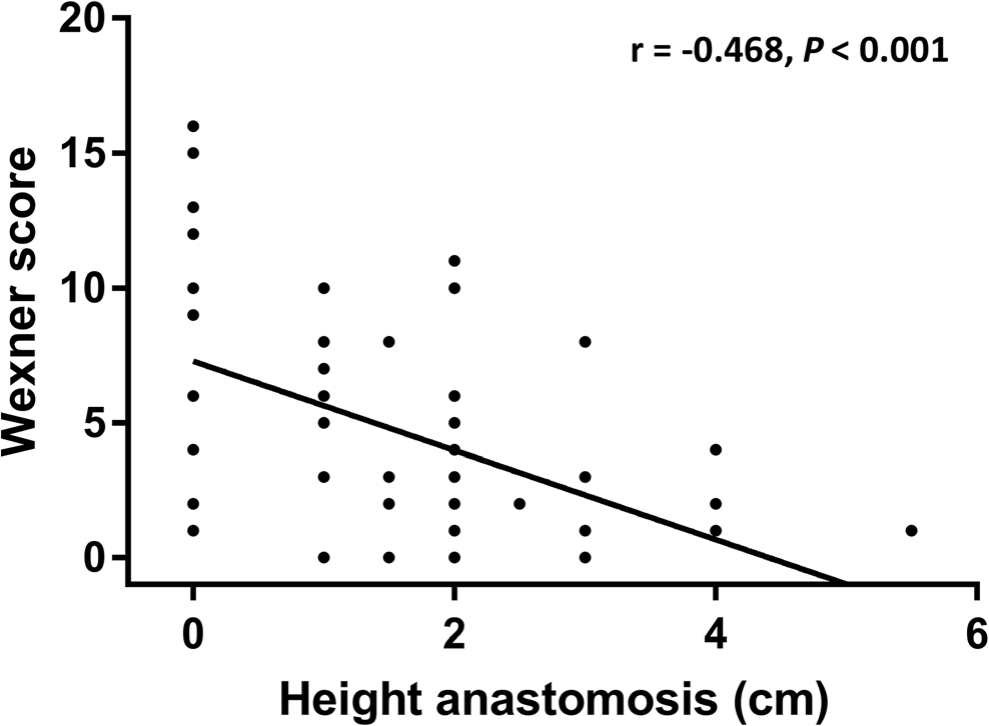
Scatterplot of the Wexner score and the height of the ileal pouch-anal anastomosis in centimeters

Patients who had undergone hand-sewn anastomosis were more likely to suffer overall fecal incontinence (OR, 3.9; 95% CI, 1.1–15), were more likely to be incontinent for liquid stool (OR, 7.7; 95% CI, 1.8–32), and were more likely to experience soiling (OR, 4.3; 95% CI, 1.1–17). Accordingly, patients with hand-sewn anastomosis had significantly higher median Wexner scores compared with patients with stapled anastomosis (10 (1–16) versus 3 (0–17), *P =* 0.003, Fig. 5). Lower height of IPAA was significantly correlated with increasing Wexner scores (*r* = − 0.468, *P* < 0.001, Fig. 6). The anastomotic height did not differ between laparoscopic surgery or open surgery (1.5 versus 2, *P =* 0.98) nor did it differ between women and men (1 versus 2, *P =* 0.21).

### Association of the IPAA stages with fecal continence outcomes

With the three-stage procedure as reference category, univariate linear regression showed that the two-stage procedure with diverting ileostomy was significantly associated with increased Wexner scores (*B* = 1.042, *P =* 0.008), while the two-stage procedure without diverting ileostomy only showed a trend towards increased Wexner scores (*B* = 0.727, *P =* 0.06). Because we found that sex and type of anastomosis influenced the patients’ Wexner scores independently, we performed multiple linear regression to correct for these parameters. Now, just the opposite was found. The two-stage procedure without diverting ileostomy showed a significant association with increased Wexner scores compared with the three-stage procedure (*B* = 0.815, *P =* 0.02), adjusted for sex (*P =* 0.008), and type of anastomosis (*P =* 0.002). The two-stage procedure with diverting ileostomy was not significantly associated anymore when we corrected for these parameters (*B* = 0.582, *P =* 0.11). The total number of the accumulated complications was comparable for all stage procedures (*P =* 0.52). There was no correlation between accumulated complications and Wexner scores (*r* = 0.074, *P =* 0.60).

## Discussion

We found that patients’ Wexner scores increased along with decreasing height of the IPAA, which means that more distal anastomoses were associated with poorer fecal continence outcomes. Moreover, patients who had undergone mucosectomies followed by hand-sewn anastomoses at the level of the dentate line had poorer outcomes regarding fecal continence compared with patients with stapled anastomoses. Although general consensus is lacking, these findings do corroborate with many previous studies, possibly thanks to better preservation of patients’ anal canals and/or anal transition zones [9–13, 29]. Interestingly, the present study showed that a longer rectal cuff, with the possibility of persistent inflammation did not have a negative effect on fecal continence outcomes. Regarding patients’ quality of life, especially the scales for general health and vitality were diminished in patients who suffered from incontinence. Soiling had the highest impact on the quality of life of patients with IPAA, with five scales diminished. The overall quality-of-life scores were comparable with previous research that also used the RAND-36 questionnaire [6, 7].

Another factor investigated in the literature is the stage procedures during IPAA. Although there is a tendency to perform a smaller number of operations, there is no consensus about which number of stages is the best option. In the current study, we found that patients who had undergone a three-stage procedure had lower Wexner scores, thus better fecal continence outcomes, even if we considered postoperative complications. Recent studies corroborate these results and report less postoperative morbidity following the three-stage procedure or when delayed pouch anastomosis was performed [15, 20, 22, 30]. Moreover, a systematic review described that proctocolectomy without diverting ileostomy is associated with an increased risk of anastomotic leakage, 9 compared with 4% [31]. Contrarily, a multicenter study showed that anastomotic leakage was similar, around 17%, for the procedure with and without an ileostomy [18]. To summarize, there is no consensus in the literature on whether the use of an ileostomy results in less anastomotic leakage. In the current study, we also found similar anastomotic leakage rates for the different stage procedures, nevertheless, we found a lower anastomotic leakage rate of 7%. Unfortunately, due to our small sample size and low event rate, we could not analyze anastomotic leakage rate in more detail by means of a multivariate analysis. Eventually, with similar anastomotic leakage rates but better fecal continence outcomes, the three-stage procedure seems most profitable for IPAA patients. We postulate that if IPAA is performed with ileostomy, tissue has the opportunity to heal with less inflammation and less fibrosis, and consequently, results in better functional outcomes for the IPAA patients. Furthermore, our conservative approach in a low-volume center for IPAA, where the three-stage procedure in the case of ulcerative colitis is standard policy, is at least as safe as in high-volume centers.

When performing a three-stage procedure, patients undergo more operations, which might be less cost effective than if less procedures were performed. Indeed, one study reported better postoperative outcomes for the three-stage procedure, while they recommended the two-stage procedure on account of the increased costs in case of the third operation [16]. From this economic point of view, a study showed that omitting an ileostomy resulted in a cost reduction of around US$10,000 per procedure, possibly because patients’ stay in hospital was reduced [32]. Conversely, permanent fecal incontinence also implies increased medical costs. A study from the Netherlands calculated that the average cost per fecal incontinent patient per year amounts to EUR2169 (equivalent to approximately US$2420 today) [33]. Considering the relatively young age of IPAA patients, the costs of fecal incontinence probably exceeds the costs of the extra procedure. Moreover, fecal incontinence constitutes a major impact on quality of life, causing difficulties for patients at work, causing patients to venture outside less often, and burdens patients’ social relationships severely [1]. Altogether, there are advantages and disadvantages of the different stage procedures. Nevertheless, the three-stage procedure seems to be the most beneficial for IPAA patients, even when taking complications and extra surgical expenses into account.

### Influence of age and sex

We found that patients who were older at the time of IPAA surgery had higher Wexner scores, while the time between surgery and questionnaire did not influence their Wexner scores. This finding indicates that although older patients have a higher risk of poor fecal continence outcomes, these outcomes do not seem to deteriorate during follow-up. We did not compare the results longitudinally, so we are unable to draw robust conclusions. Nevertheless, our findings corroborate previous research that reported a stable situation even after approximately 15 years [34, 35].

The current study showed a lower Wexner score in men compared with women. The literature also reports better fecal continence outcomes for men with IPAA compared with women [30]. This is the reason for our adjustment for sex in the multiple linear regression analysis.

### Limitations

Our results might be biased because, naturally, we could not send questionnaires to patients with an inactive pouch and who had died. Consequently, we could not include an analysis of the fecal continence outcomes of these patients. Nevertheless, patient characteristics were similar between the group who had completed the questionnaire and the group who had not, except that the age of the questionnaire group at IPAA surgery was higher. The current study also showed that a higher age at IPAA surgery resulted in poorer fecal continence outcomes. We may therefore perhaps have presented poorer outcomes than if all our IPAA patients, including the younger ones, had participated. It is possible that the younger patients did not respond to the questionnaire because they did not have any fecal complaints. Nevertheless, it is also known that younger individuals are less willing to fill out questionnaires. A second limitation of this study is the small number of patients over a long period of time. A third limitation is that there were more patients with S-pouches who underwent mucosectomy. Nevertheless, we expect no bias of our incontinence outcomes, as patients with S-pouches tend to suffer from outlet obstructions [36, 37]. Finally, a limitation of this study is that the height of the anastomoses was estimated subjectively rather than with a ruler to indicate the exact height in centimeters. We did record whether the anastomoses were hand-sewn or stapled, thus whether the anastomosis was created at the dentate line (0 cm) or higher. This more accurate parameter also showed a benefit for the proximal anastomoses.

## Conclusions

Quality of life was reduced in patients who had undergone proctocolectomy with IPAA and who suffered from fecal incontinence. Soiling had the highest impact on the quality of life. Patients who had undergone mucosectomy followed by a hand-sewn anastomosis experienced poorer fecal continence outcomes compared with patients with a stapled anastomosis. They were also more likely to be incontinent for liquid stool and experienced more soiling. Patients who were older at the time of IPAA surgery experienced poorer fecal continence outcomes, while the time between surgery and questionnaire did not influence their continence outcomes. Additionally, more distal IPAA anastomoses were associated with poorer fecal continence outcomes. IPAA performed in a three-stage procedure seems to give the best clinical results, even when taking complications into account.

## Data Availability

Upon reasonable request.
